# Diagnosis of diabetes in pregnant woman using a Chaotic-Jaya hybridized extreme learning machine model

**DOI:** 10.1515/jib-2019-0097

**Published:** 2020-08-13

**Authors:** Prajna Paramita Debata, Puspanjali Mohapatra

**Affiliations:** Department of Computer Science and Engineering, International Institute of Information Technology, Bhubaneswar, Odisha, India

**Keywords:** Chaotic Jaya algorithm, diabetes diagnosis, extreme learning machine, multi-layer perceptron, optimization, teaching learning based optimization

## Abstract

As stated by World Health Organization (WHO) report, 246 million individuals have suffered with diabetes disease over worldwide and it is anticipated that by 2025 this estimation can cross 380 million. So, the proper and quick diagnosis of this disease is turned into a significant challenge for the machine learning researchers. This paper aims to design a robust model for diagnosis of diabetes using a hybrid approach of Chaotic-Jaya (CJaya) algorithm with Extreme Learning Machine (ELM), which is named as CJaya-ELM. In this paper, Jaya algorithm with Chaotic learning approach is used to optimize the random parameters of ELM classifier. Here, to assess the efficacy of the designed model, Pima Indian diabetes dataset is considered. Here, the designed model CJaya-ELM, has been compared with basic ELM, Teaching Learning Based Optimization algorithm (TLBO) optimized ELM (TLBO-ELM), Multi-Layer Perceptron (MLP), Jaya algorithm optimized MLP (Jaya-MLP), TLBO algorithm optimized MLP (TLBO-MLP) and CJaya algorithm optimized MLP models. CJaya-ELM model resulted in the highest testing accuracy of 0.9687, sensitivity of 1, specificity of 0.9688 with 0.9782 area under curve (AUC) value. Results reveal that CJaya-ELM model effectively classifies both the positive and negative samples of Pima and outperforms the competitors.

## 1 Introduction

Diabetes is dreadful for human beings as it threatens them irrespective of their age and gender. It is not a disease caused by any pathogens, but the deficiency of insulin. However, its impact is so harmful upon vital organ that it is regarded as a mother of all diseases. The impact of diabetes is worst on women in comparison to men due to lower longevity rate and substandard condition of life. According to World Health Organization (WHO) reports, majority of the women who are affected by diabetes haven’t any information about it. Especially in case of pregnant women, this disease can be transmitted to their offspring. In case of diabetic women, they are suspectable to miscarriage, kidney failure, heart strokes, blindness and other chronic and fatal ailments [1]. Due to this purpose, a faster diagnosis of diabetes in case of pregnant woman is very much essential.

Normally, a person is said to be diabetic, if his/her blood sugar level is above the range of 4.4–6.1 mmol/L [1]. Generally, less hormone production or no proper insulin production occurs in diabetic patients. Three types of diabetic patients are often seen, which are Gestational, Type 1 and Type 2 [2]. Due to the damage of insulin secretion of pancreatic cells, Type 1 diabetes occurs at early ages in the cases of teens which is termed as autoimmune disease. Type 2 diabetes occurs when the different parts of the body become immune to insulin and pancreas is not able to produce the required amount of insulin. Pregnant women generally suffer from Gestational diabetes when the pancreas cannot be able to make the needed quantity of insulin. All the complicacy due to diabetes can be avoided, if it is diagnosed at an early stage.

Since a decade back it is of the top challenges for machine learning researchers to diagnose diabetes. Iyer et al. [3] applied classification mining methods for the identification of diabetes. T. Santhanam et al. [4] used K-Means with genetic algorithms for dimensionality reduction of diabetes data and applied Support Vector Machine (SVM) for classifying the diabetes data. Kavakiotis et al. [5] established the link between machine learning approaches and diabetes research. R Gargeya and T Leng [6] introduced automatic detection of diabetes by applying a deep learning approach. Md.Maniruzzaman et al. [7] proposed a comparative method of diabetes data classification by employing a machine learning model. H. Kaur et al. [8] introduced a predictive model for diabetes by implementing a machine learning technique. R. F. Mansour et al. [9] applied a deep learning for automatic diagnosis of diabetes. S. Perveen [10] developed a predictive model for identifying diabetes using machine learning techniques. Siva Shankar G. et al. [11] introduced an optimized fuzzy rule based grey wolf optimization algorithm for identifying diabetes.

Still it is challenging to handle the complexity of diabetes after a variety of studies on designing better classifiers. As a result, scopes for solving such problem are always open. So far, many statistical classifiers as well as many soft computing techniques have been used successfully for classification. Some of them are: Multilayer perceptron (MLP) [12], Bayesian decision theory [13], Euclidean minimum distance (EMD) [14], k-nearest neighbour (KNN) classifiers [15], fuzzy rule-based systems [16], SVM [12], [13], [14], [17] and Back-Propagation (BP) [18] classifiers. These traditional learning approaches suffer from large number of shortcomings like: as trapping at local optima, unvarying learning rate and adjustment of random parameters [19]. To deal with these drawbacks Huang et al. introduced extreme learning machine (ELM) [20] algorithm. This is otherwise known as generalized single-hidden layer feed forward networks (SLFNs). In spite of sensible generalization ability with quicker learning speed, ELM has some limitations [21], [22]. The choice of a better activation function and random parameters in this classifier may produce the unstable solutions. While solving classification and regression problems, these random parameters may create uncertainty. To minimize the training error rate, the output weights of ELM are estimated from randomly chosen input weights and hidden biases [23]. For the optimum selection of random parameters, the ELM model is optimized with various meta-heuristic learning algorithms viz. evolutionary algorithms, swarm intelligence and other nature inspired algorithms. From evolutionary learning algorithms, genetic algorithm (GA) hybridised ELM [24] as well as differential evolution (DE) hybridised ELM [25] have already been successfully used for the classification of medical data. In swarm intelligence algorithms, ELM optimized with Particle Swarm Optimization (PSO) algorithm [26] has been effectively applied. From other nature inspired algorithms like Cuckoo Search (CS) [27] and Cat Swarm Optimization (CSO) hybridised ELM [28] have been successfully applied by the researchers.

In this study, a recently developed Jaya optimization algorithm [29] is applied to optimize random parameters of ELM. This algorithm is introduced by R. Venkata Rao which is able to handle both unconstrained and constrained optimization problems. It is designed on the basis of keeping the best one and removing the worst one. Recently, many researchers [30], [31], [32], [33] have applied this algorithm for solving different problems. As Jaya algorithm is working with two random variables it may produce suboptimal result. To deal with this problem, Chaos theory is integrated with Jaya algorithm. The random numbers of Jaya algorithm are generated by adopting a chaotic random number generator which not only produces optimal result but also improves the convergence speed and provides the better exploration of the search space without trapping in local optima. In this work, Chaos theory upgraded Jaya algorithm [34] is hybridised with ELM and designs a robust classifier, called as the Chaotic Jaya-ELM (CJaya-ELM) model.

The remaining part of the study is structured as follows: the model description part is described in section 2, section 3 focuses on all the methodologies related to this study, section 4 enlightens all the experimental part, section 5 describes the results analysis part and section 6 is ended with the conclusion part.

## 2 Model description

**Figure 1:**
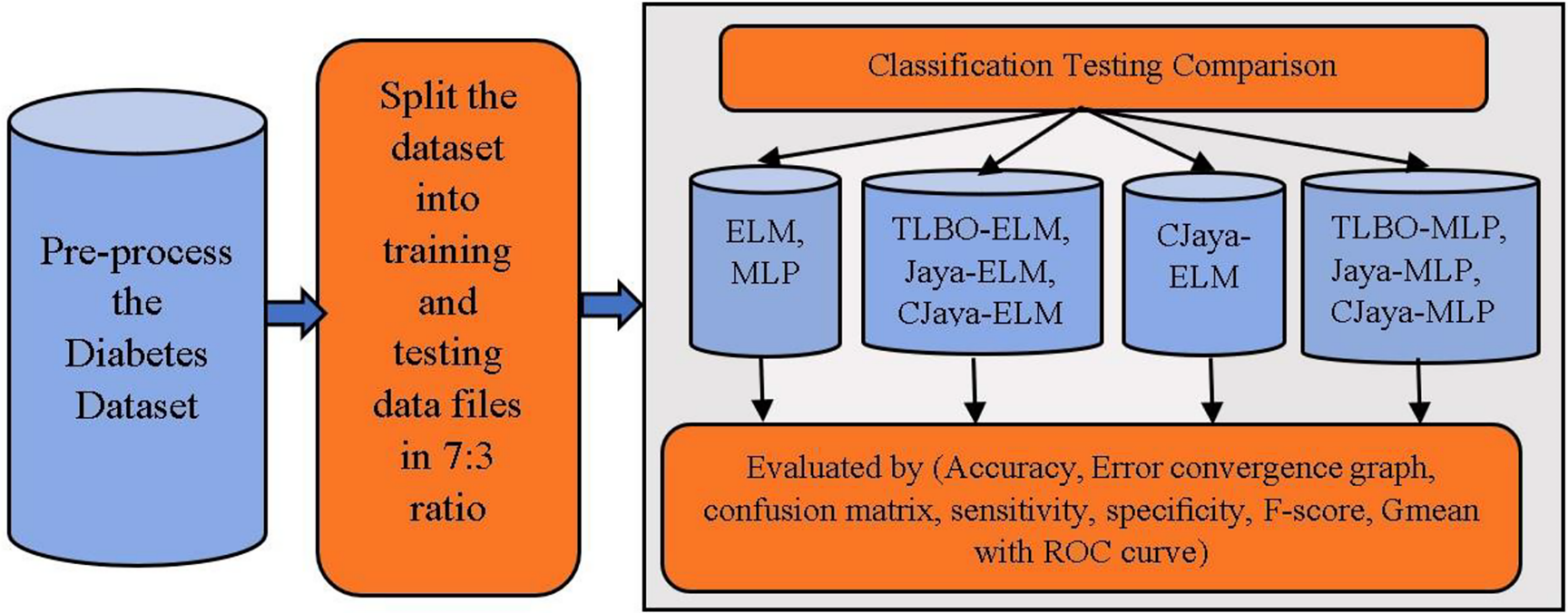
Overall system architecture of diabetes data classification.

The overall architecture of diabetes data classification model is depicted in Figure 1. Here, Pima Indian diabetes dataset is considered to test all the models. The source of this dataset is UCI repository [35]. The attributes of this dataset contain the following information of a pregnant woman such as the number of times a woman is pregnant, concentration of glucose, thickness of skin fold, blood pressure rate, insulin rate, body mass index (BMI) and diabetes pedigree function including patient’s age.

The detail clarification about this dataset is given in Table 1. Here, min–max normalization is used to normalize the dataset in the pre-processing phase by applying Eq. (1) within the range (−1, 1).

(1) $$M_{n}=a+\left(b-a\right)\times \frac{M-D_{\text{min}}}{D_{\text{max}}-D_{\text{min}}}$$

**Table 1:** Description of the Pima Indian diabetes datasets.

Dataset	Samples size	Features size including class label	Classes	Presence of missing attributes	Presence of noisy attributes
Pima Indian diabetes	768	9	2	No	No

In Eq. (1) $M_{n}$ represents the normalized form of original value *M*, the values of *a* and *b* are taken as −1 and 1 respectively, $D_{\text{min}}$ is the minimum and $D_{\text{max}}$ is the maximum value of the dataset.

**Figure 2:**
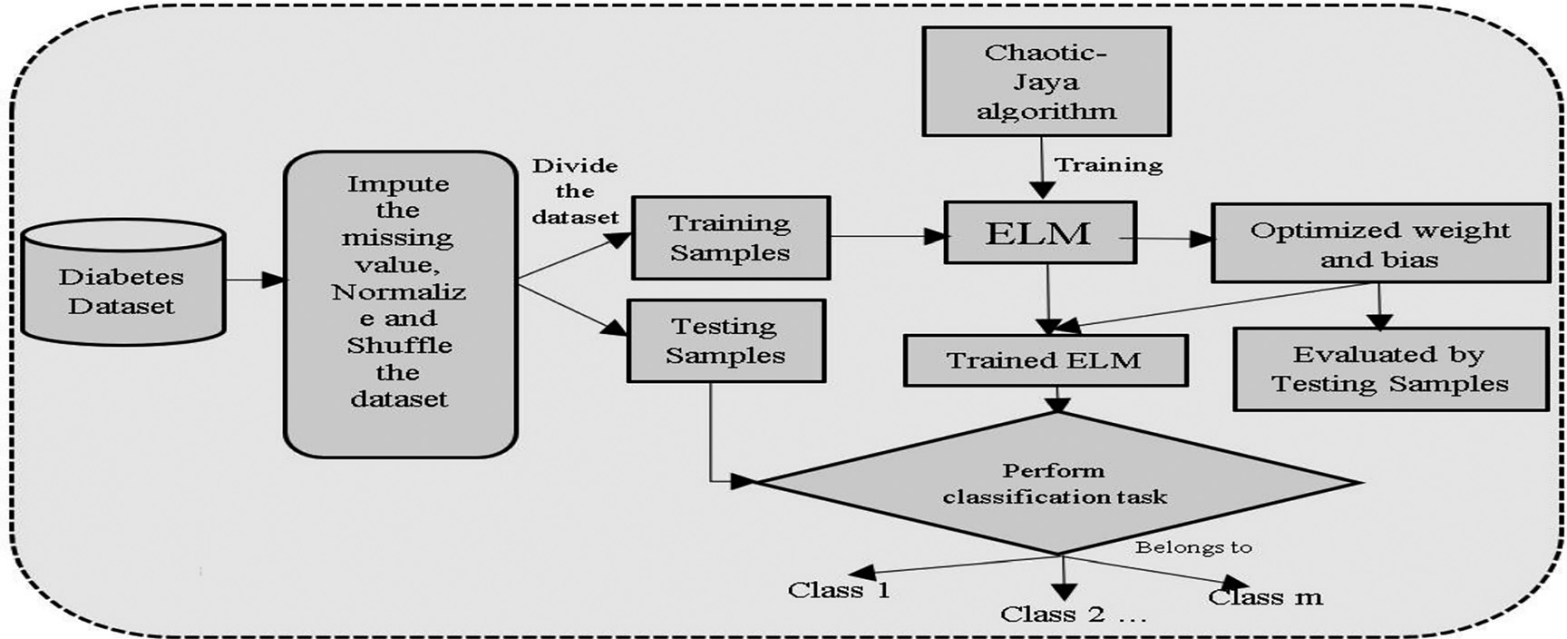
Block diagram of the proposed CJaya-ELM model.

Here, randperm() function is used to shuffle the dataset. After shuffling, the diabetes dataset is separated into training file and testing file, in the 7:3 ratio. In the training phase, this model is trained using CJaya-ELM, Jaya-ELM, TLBO-ELM, basic ELM, MLP, Jaya-MLP, TLBO-MLP, and CJaya-MLP algorithms. The performance of this model is estimated by classification accuracy percentage, confusion matrix, specificity, sensitivity, F-score, Gmean including ROC graph with area under curve (AUC) values. In proposed model, the training samples are fed to ELM classifier which is trained by CJaya algorithm and the trained ELM performs the classification task efficiently. Here, the CJaya algorithm optimizes the randomly taken weights and biases of ELM which can create non-optimal results. The trained CJaya-ELM model is tested by testing samples and calculated results are obtained. When the calculated results are compared with the target value, it represents the misclassification rate. The Overall schema of the presented CJaya-ELM model is given below in Figure 2.

## 3 Supported methodologies

This section discusses about all the supported methodologies, used in this study.

### 3.1 ELM model

**Figure 3:**
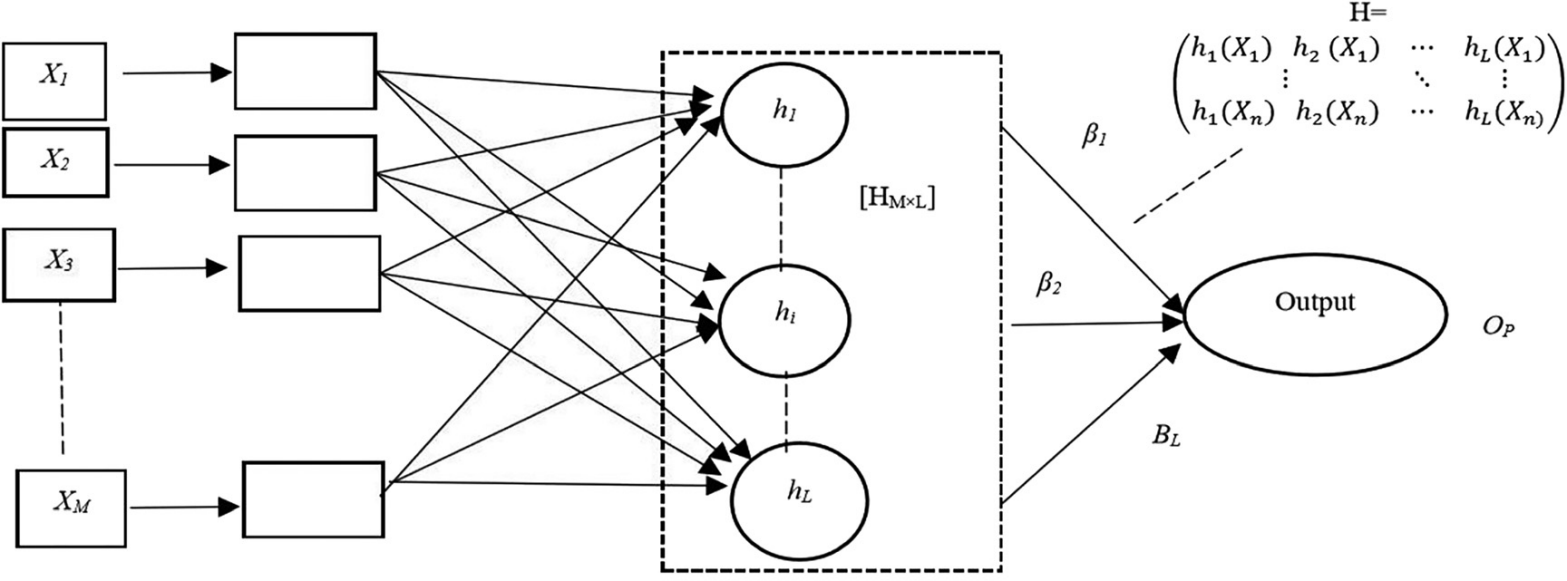
The basic architecture of ELM.

ELM has high significance as compared to other neural networks because it is computationally free from iterations which makes its learning speed faster. The basic architecture of ELM is depicted in Figure 3.

The steps of basic ELM algorithm are given below in a summarized manner:

1. Randomly pick the input weights (*w*_*i*_) and hidden layer biases (*b*_*i*_).
2. Determine the output matrix (*H*) of the hidden layer.
3. Compute the output weight ($\hat{\beta }$) using the equation Eq. (2)
  (2) $$\hat{\beta }=H^{†}T$$
  Here, *T* is the target value.

In the ELM, the input weights and biases in the hidden layer are chosen randomly and the output weights are calculated accordingly. However, it causes two problems. First one is, ELM may need more neurons in comparison to traditional [20] tuning based machine learning algorithms and it concludes that ELM responds slow for unknown testing data. The second one is that, when large number of hidden layer neurons are used, an ill-conditioned [20] hidden output matrix (H) may be formed which may deteriorate the generalization performance. ELM is not required to be tuned like SLFN. The primary attraction of ELM is that it calculates the output weights, rather than tuning the hidden layer. In this work, the performance of ELM models is compared with MLP classification models, which is widely accepted for solving real life problems.

### 3.2 MLP model

The MLP [27], [36] classifier is designed with multiple layers of fully connected neurons. The neurons interact with each other by weighted connections. Basically, in MLP, any number of hidden layers may exist in the middle of input layer and output layer. Here, a three hidden layer MLP model is considered, where each hidden layer contains five nodes. In the input layer, the input vector, {Y_1_, Y_2_, … Yn} is given to the model. The expected output is provided by the supervisor. If an MLP model consists of two hidden layers, then the total input, $Y_{j}^{L_{2}}$ received by *j*th neuron in the hidden layer $L_{2}$ is interpreted as Eq. (3)

(3) $$Y_{j}^{L_{1}}=\underset{i}{\sum }n_{i}^{L_{1}}w_{ji}^{L_{1}}$$

where $n_{i}^{L_{1}}$ depicts the *i*th neuron of the previous hidden layer $L_{1}$, $w_{ji}^{L_{1}}$ represents the weight of the link from the *i*th neuron in the hidden layer $L_{1}$ to the *j*th neuron in the hidden layer $L_{2}$.

The output of a neuron is shown as a nonlinear sigmoid activation function of its total input and is defined as Eq. (4)

(4) $$n_{j}^{L_{1}}=\frac{1}{1+e^{-Y_{j}^{L_{1}}}}$$

In the input layer, the outputs of all nodes are defined in Eq. (5)

(5) $$n_{j}^{0}=Y_{j}^{0}$$

where $Y_{j}^{0}$ is the *j*th component of the input vector in the input layer.

In MLP, BP learning algorithm is used to find out all internal weights of the hidden units. The error related to weight vector ‘*w*’ and output vectors, is calculated by Least Mean Square (LMS) error calculation method using the Eq. (6)

(6) $$E\left(w\right)=\frac{1}{2}\underset{j,s}{\sum }{\left(n_{j,s}^{L}\left(w\right)-d_{j,s}\right)}^{2}$$

Here, $n_{j,s}^{L}$ is the output for node *j* in *L*th layer of *s*th input/output case and *d*_*j,s*_ is taken as the desired output. To minimize *E(w)* the gradient-descent method is used and a sequence of weight updating are carried out by applying the formula of Eq. (7)

(7) $$\Delta w_{j,i}^{L_{1}}\left(t\right)=-\varepsilon +\Delta w_{j,i}^{L_{1}}\left(t-1\right)$$

Here, *ε* is defined as a positive constant, 0≤α≤1 defines the coefficient of momentum.

**Figure 4:**
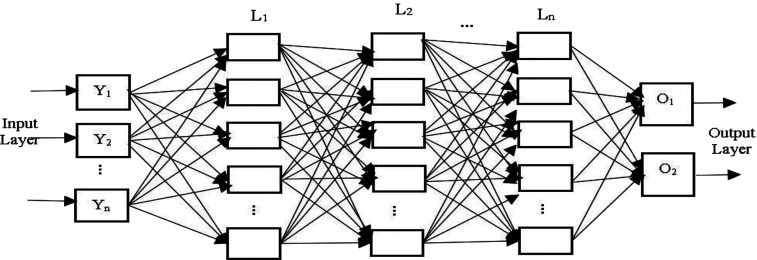
The basic architecture of MLP.

Moreover, to deal with the local minima problem of BP algorithm, MLP is integrated with Jaya and CJaya, Teaching Learning Based Optimization algorithms (TLBO) and the outcomes are compared with ELM based models. The basic architecture of MLP model is shown in Figure 4.

### 3.3 TLBO algorithm

TLBO is used to deal with non-linear optimization problem. This algorithm is influenced by teaching learning process. TLBO algorithm [37] is based on two phase operations: (a) Teacher Phase and (b) Learner Phase. The steps of the TLBO algorithm is shown below:

1. Input the number of population and stopping condition.
2. Get the mean of the design variable.
3. Initialize the teacher as the best solution.
4. Change the solution using the Eq. (8):
  (8) $$Z_{\text{new}}=Z_{\text{old}}+r\left(Z_{\text{teacher}}-\left(T_{f}\right)\text{Mean}\right)$$
5. Check, if the existing one is better than the new one, then reject the new one. Else, accept the new solution and pick any two solutions $\left(Z_{i},\;Z_{j}\right)$ arbitrarily.
6. If *Z*_*i*_ is better than *Z*_*j*_, then apply Eq. (9)
  (9) $$Z_{\text{new}}=Z_{\text{old}}+r\left(Z_{i}-Z_{j}\right),\;\text{else}\;Z_{\text{new}}=Z_{\text{old}}+r\left(Z_{j}-Z_{i}\right)$$
7. If the existing one is better than $Z_{\text{new}}$, then reject the solution, otherwise accept the solution and check the stopping condition.
8. If the stopping condition is satisfied, then get the final solution. Otherwise, repeat the procedures from step 2 to step 7.

### 3.4 Jaya optimization algorithm

Like TLBO, Jaya is another optimization [29] approach which does not need any specific algorithm-oriented parameters. It requires less computational time, less implementation complexity with faster convergence rate than TLBO algorithm. The steps of Jaya algorithm are elaborated below:

1. Set the size of the population, number of design variables and the stopping condition.
2. Get the best and worst solutions from the population.
3. The result according to the best and worst solutions would be changed by applying Eq. (10).
  (10) $$Z_{j,k,i}^{\prime }=Z_{j,k,i\;}+r_{1,j,i}\left[\left(Z_{j,best,i}\right)+\left|\left(Z_{j,k,i}\right)\right|\right]-r_{2,j,i}\left[\left(Z_{j,worst,i}\right)-\left|\left(Z_{j,k,i}\right)\right|\right]$$
  During *i*th iteration, $Z_{j,k,i}$ is the value of the *i*th variable for the *k*th candidate. Here, *k* is the population size, *i* is the number of iteration and *j* is the number of design variables.
4. Then the existing solution is compared with the modified one and if it is found that the modified solution is better, then it will be exchanged by the previous one, else the previous solution will be kept.
5. The procedures, from step 2 to step 4 will be repeated till the stopping condition is reached.

### 3.5 Chaotic learning method

In this work, one of the variants of Jaya algorithm, Chaotic-Jaya (CJaya) is used. This algorithm is established on the chaos theory. This algorithm improves the convergence speed and provides the better exploration of the search space without trapping in local optima [38], [39]. In mathematical term, Chaos is defined as the randomness of a deterministic dynamical system. To interpret chaos theory in different optimization algorithms, various chaotic maps with various mathematical equations are applied. In this work, from various functions, logistic map function is used for generating the chaotic random numbers due to its simplicity and it is defined by Eq. (11).

(11) $$x_{t+1}=4x_{t}\left(1-x_{t}\right)$$

where *x*_*t*_ is the obtained value of the chaotic map at *t_th_* iteration.

The working principle of the CJaya algorithm is same as the Jaya algorithm. The main difference is that, the random numbers in CJaya algorithm are generated by adopting a chaotic random number generator. Here, the two random variables (*r*_1_ and *r*_2_) of Jaya algorithm are substituted by the logistic chaotic variables. The population is updated as Eq. (12).

(12) $$Z_{j,k,i}^{\prime }=Z_{j,k,i}+x_{t,j,i}\left[\left(Z_{j,best,i}\right)+\;\left|\left(Z_{j,k,i}\right)\right|\right]-x_{t,j,i}\left[\left(Z_{j,worst,i}\right)-\left|\left(Z_{j,k,i}\right)\right|\right]$$

Here, t represents the iteration number, *x*_*t*_ is the value of *t_th_* chaotic iteration, and the initial value of *x*_0_ is randomly created in between [0, 1].

### 3.6 Proposed CJaya-ELM algorithm

**Algorithm:**

Pima diabetes data classification by using CJaya-ELM model.

*Input*: Pima diabetes dataset; Population size (PS); Hidden layer size (*H*_*c*_)

*Output*: Classification accuracy

*Description*: *newP*_*i*_ is the new population, *Obj*_*i*_ is the objective value of the new population, *W* is the weight vector, *train_acc_percentage* and *test_acc_percentage* are accuracy percentage of *train_data* and *test_data* respectively

1. Split the dataset into train_data (*training_input, training_output*) and test_data (*testing_input, testing_output*) in the ratio of 7:3
2. Generate *H*_*c*_ no. of random weight population, each having size of 1 × *H*_*c*_ $P_{i}=\left\{W_{1}^{i},W_{2}^{i},W_{3}^{i},\ldots ,W_{H_{c}}^{i}\right\}$ for *i* = 1,2,3, …PS
  Find the values of the chaotic map *x*_*m*_, by applying Eq. (11)
  Update the two random values *r*_*1*_ and *r*_*2*_ in Eq. (10) using Eq. (11)
3. For each population *P*_*i*_, find the error value or *miss_classification_rate* in ELM by Step 4 to Step 8
4. For each *training_input* find
5. *H = training_input* × *P*_*i*_
6. *β*_*i*_ *= pseudo_inverse* × *training_output*
7. *obtained_output* = (*testing_input* × *P*_*i*_) × *βi*
8. *Obj*_*i*_ is calculated from *miss_classification_rate* which is estimated by comparing *obtained_output* and *testing output*
9. Find *g_best P*_*i*_ where *argmin*(*Obj*_*i*_)
10. Find *g_worst P*_*i*_ where *argmax*(*Obj*_*i*_)
11. For each population *P*_*i*_, find *newP*_*i*_ using Eq. (12).
12. Find *nObj*_*i*_ by Step 4 to Step 8
13. If *Obj*_*i*_>*nObj*_*i*_
14. Replace *P*_*i*_ with *new P*_*i*_
15. Repeat Step 9 to Step 14, till the termination criteria reaches
16. *g_best* is considered as the final weight *W*_*g_best*_ and keep the corresponding *β*_*g_best*_
17. *Calculated_output* = (*testing_input* × *W*_*g_best*_) × *β*_*g_best*_ and determine the actual label of the class for both train_data and test_data
18. Determine the classification accuracy by comparing desired label and actual label of the class

### 3.7 Performance measuring attributes

Several performance measuring attributes are taken in this study, such as accuracy percentage, specificity, sensitivity, Confusion matrix, Gmean and F-score. Confusion matrix evaluation method is applied, when there are two possible outcomes. Confusion matrix consists of True Positive (TP), True Negative (TN), False Positive (FP), and False Negative (FN) values [24]. The detail descriptions about these methods are given in the Table 2.

**Table 2:** Detail descriptions about the performance measures.

Performance measures	Description
TP	When the positive samples are classified accurately
TN	When the negative samples are classified accurately
FP	When the negative samples are misclassified
FN	When the positive samples are misclassified
Accuracy	It is the overall classification accuracy percentage resulted by a standard classifier.
Sensitivity (Sen)	It determines the proportion of true positive samples in total samples and called as True Positive Rate (TPR) Sn=TP/(TP+FN)
Specificity (Spe)	It identifies the proportion of true negative samples in total samples and called as False Positive Rate (FPR) Sp=TN/(TN+FP)
Gmean	It is determined from both, specificity and sensitivity. Gmean = $\sqrt{\text{Sp}\times \text{Sn}}$
F-score	F-score value gives the combined performance of the two classes F-score = (2 × Sp × Sn)/(Sp + Sn)
ROC	It is a graphical representation between sensitivity and specificity. ROC plots TPR versus FPR at different classification thresholds.
AUC	It is an aggregate evaluation of performance across all possible classification thresholds. So, it is called as Area under ROC curve.

## 4 Experimental analysis

Here, the experimental analysis section is separated into two phases. The first phase presents the miss classification rate w.r.t number of iterations graph of different models which gives a clear idea about the convergence speed of different algorithms. The second phase contains training accuracy, testing accuracy, training time, TP, TN, FP, FN values, specificity, sensitivity, F-score, Gmean, and ROC with AUC values of all the ELM based models. The results of the proposed model are observed from 10 to 300 number of hidden nodes with each run there is an increment of 10 neurons. In this study, the value of population size and the number of iterations is set as 100. Among different activation functions, sigmoidal function is taken for all the models in this study.

All the experiments of this work are implemented in an environment having Windows 10 operating System, Intel(R) Core (TM) i5-7200U CPU of 2.5 GHz processing speed with 8 GB RAM. Language used in this work is MATLAB (version: R2015b, 64 bits).

### 4.1 Phase I: description of miss classification rate convergence graph

**Figure 5:**
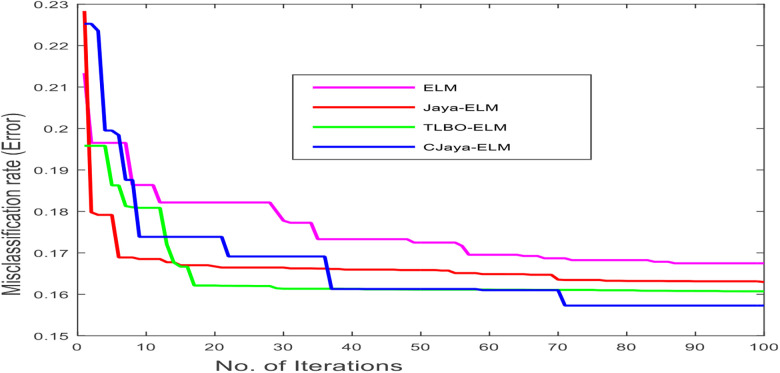
No. of iterations versus miss classification rate of ELM based models.

The main purpose of the proposed CJaya-ELM algorithm is to improve the convergence speed and providing the better exploration of the search space without trapping at local optima. In this work, ELM is taken as an objective function. Here, the global best is considered as the minimum objective value (miss classification rate) whereas the global worst is considered as the maximum objective value (miss classification rate). At first iteration, the weights and biases are chosen arbitrarily, after that the weight is modified using the optimization technique and this process repeats up to the end of iterations. The error or miss classification rate convergence graph of ELM, TLBO-ELM, Jaya-ELM and CJaya-ELM is shown in Figure 5, where it is clearly visualised that the converging rate of the proposed CJaya-ELM model is better than other models.

### 4.2 Phase II: evaluating all the models by various performance measuring variables

In this phase, all the ELM based models such as ELM, TLBO-ELM, Jaya-ELM and CJaya-ELM are evaluated by training and testing accuracy, specificity, sensitivity, confusion matrix, Gmean, F-score, and ROC with AUC values. The highest testing accuracy of all MLP based models and ELM based models is also compared in this study. The training with testing accuracy for ELM, TLBO-ELM, Jaya-ELM and CJaya-ELM appear in Table 3. In all the tables, HN stands for the size of hidden neurons. Table 4 shows the sensitivity, TP, TN, FP and FN values for ELM, TLBO-ELM, Jaya-ELM and CJaya-ELM. The specificity, F-score and Gmean values of all the ELM based models are demonstrated in Table 5.

**Table 3:** Training accuracy (Tr_Acc) and Testing accuracy (Ts_Acc) for ELM, TLBO-ELM, Jaya-ELM and CJaya-ELM.

NH	ELM		TLBO-ELM		Jaya-ELM		CJaya-ELM	
	Tr_Acc	Ts_Acc	Tr_Acc	Ts_Acc	Tr_Acc	Ts_Acc	Tr_Acc	Ts_Acc
10	0.8794	0.8668	0.9637	0.9325	0.9641	0.9473	0.9701	0.9483
20	0.8892	0.8721	0.9629	0.9385	0.9707	0.9467	0.9727	0.9457
30	0.8990	0.8719	0.9735	0.9402	0.9705	0.9460	0.9715	0.9471
40	0.9049	0.8683	0.9741	0.9465	0.9756	0.9483	0.9736	0.9495
50	0.9107	0.8680	0.9719	0.9480	0.9766	0.9473	0.9783	0.9496
60	0.9148	0.8676	0.9753	0.9475	0.9790	0.9480	0.9791	0.9498
70	0.9198	0.8596	0.9769	0.9440	0.9784	0.9491	0.9793	0.9502
80	0.9248	0.8626	0.9763	0.9395	0.9806	0.9480	0.9815	0.9496
90	0.9297	0.8606	0.9761	0.9360	0.9810	0.9447	0.9812	0.9487
100	0.9254	0.8705	0.9793	0.9435	0.9826	0.9442	0.9843	0.9494
110	0.9318	0.8747	0.9814	0.9495	0.9850	0.9498	0.9862	0.9517
120	0.9334	0.8731	0.9839	0.9459	0.9888	0.9463	0.9889	0.9536
130	0.9407	0.8840	0.9859	0.9515	0.9868	0.9501	0.9872	0.9551
140	0.9432	0.8825	0.9891	0.9470	0.9902	0.9513	0.9912	0.9562
150	0.9508	0.8880	0.9894	0.9495	0.9918	0.9487	0.9918	0.9581
160	0.9547	0.8798	0.9923	0.9518	0.9948	0.9552	0.9953	0.9587
170	0.9586	0.8910	0.9941	0.9525	0.9952	0.9547	0.9969	0.9592
180	0.9621	0.8936	0.9963	0.9575	0.9966	0.9581	0.9971	0.9619
190	0.9625	0.8978	0.9969	0.9530	0.9970	0.9567	0.9987	0.9595
200	0.9638	0.9045	0.9983	0.9515	0.9994	0.9547	0.9996	0.9617
210	0.9664	0.9068	0.9980	0.9580	0.9998	0.9593	0.9998	0.9633
220	0.9714	0.9161	0.9993	0.9591	0.9998	0.9603	**1**	0.9627
230	0.9723	0.9171	**1**	0.9570	**1**	0.9627	1	**0.9687**
240	0.9732	0.9189	1	0.9585	1	0.9633	1	0.9643
250	0.9749	0.9196	1	0.9565	1	0.9647	1	0.9658
260	0.9768	0.9214	1	0.9612	1	**0.9680**	1	0.9679
270	0.9783	0.9198	1	0.9654	1	0.9663	1	0.9673
280	0.9798	0.9260	1	0.9625	1	0.9662	1	0.9652
290	0.9813	0.9289	1	**0.9660**	1	0.9666	1	0.9681
300	**0.9865**	**0.9302**	1	0.9645	1	0.9667	1	0.9678

Bold values are the highest values.

**Table 4:** Sensitivity, TP, FN, FP and TN values for ELM, TLBO-ELM, Jaya-ELM and CJaya-ELM.

HN	ELM					TLBO-ELM					Jaya-ELM					CJaya-ELM				
	Sen	TP	FN	FP	TN	Sen	TP	FN	FP	TN	Sen	TP	FN	FP	TN	Sen	TP	FN	FP	TN
10	0.8727	144	21	20	45	0.9515	157	8	20	48	0.9576	158	7	23	42	0.9578	159	7	23	41
20	0.8688	139	21	13	57	0.9500	152	8	13	60	0.9563	153	7	16	54	0.9563	153	7	16	54
30	0.8766	135	19	18	58	0.9610	148	6	18	61	0.9675	149	5	21	55	0.9677	150	5	21	54
40	0.8896	137	17	23	53	0.9740	150	4	23	56	0.9805	151	3	26	50	0.9805	151	3	26	50
50	0.8841	145	19	14	52	0.9634	158	6	14	55	0.9695	159	5	17	49	0.9697	160	5	17	48
60	0.8827	143	19	22	46	0.9630	156	6	22	49	0.9691	157	5	25	43	0.9691	157	5	25	43
70	0.8792	131	18	23	58	0.9664	144	5	23	61	0.9732	145	4	26	55	0.9733	146	4	26	54
80	0.8563	143	24	20	43	0.9341	156	11	20	46	0.9401	157	10	23	40	0.9401	157	10	23	40
90	0.8627	132	21	18	59	0.9477	145	8	18	62	0.9542	146	7	21	56	0.9545	147	7	21	55
100	0.8408	132	25	20	53	0.9236	145	12	20	56	0.9299	146	11	23	50	0.9299	146	11	23	50
110	**0.9108**	**143**	**14**	**24**	**49**	**0.9936**	**156**	**1**	**24**	**52**	**1**	**157**	**0**	**27**	**46**	**1**	**158**	**0**	**27**	**45**
120	0.9000	135	15	24	56	0.9867	148	2	24	59	0.9933	149	1	27	53	0.9933	149	1	27	53
130	0.8735	145	21	14	50	0.9518	158	8	14	53	0.9578	159	7	17	47	0.9581	160	7	17	46
140	0.8247	127	27	29	47	0.9091	140	14	29	50	0.9156	141	13	32	44	0.9156	141	13	32	44
150	0.8683	145	22	17	46	0.9461	158	9	17	49	0.9521	159	8	20	43	0.9524	160	8	20	42
160	0.8909	147	18	13	52	0.9697	160	5	13	55	0.9758	161	4	16	49	0.9758	161	4	16	49
170	0.8466	138	25	18	49	0.9264	151	12	18	52	0.9325	152	11	21	46	0.9329	153	11	21	45
180	0.7901	128	34	24	44	0.8704	141	21	24	47	0.8765	142	20	27	41	0.8765	142	20	27	41
190	0.8333	135	27	17	51	0.9136	148	14	17	54	0.9198	149	13	20	48	0.9202	150	13	20	47
200	0.8726	137	20	22	51	0.9554	150	7	22	54	0.9618	151	6	25	48	0.9618	151	6	25	48
210	0.8269	129	27	24	50	0.9103	142	14	24	53	0.9167	143	13	27	47	0.9172	144	13	27	46
220	0.8258	128	27	14	61	0.9097	141	14	14	64	0.9161	142	13	17	58	0.9161	142	13	17	58
230	0.8494	141	25	16	48	0.9277	154	12	16	51	0.9337	155	11	19	45	0.9341	156	11	10	53
240	0.8431	129	24	27	50	0.9281	142	11	27	53	0.9346	143	10	30	47	0.9346	143	10	20	57
250	0.7888	127	34	13	56	0.8696	140	21	13	59	0.8758	141	20	16	53	0.8765	142	20	6	58
260	0.8385	135	26	17	52	0.9193	148	13	17	55	0.9255	149	12	20	49	0.9255	149	12	10	59
270	0.8553	136	23	15	56	0.9371	149	10	15	59	0.9434	150	9	18	53	0.9437	151	9	6	64
280	0.8113	129	30	18	53	0.8931	142	17	18	56	0.8994	143	16	21	50	0.8994	143	16	4	67
290	0.8182	135	30	23	42	0.8970	148	17	23	45	0.9030	149	16	26	39	0.9036	150	16	2	62
300	0.8187	131	29	13	57	0.9000	144	16	13	60	0.9063	145	15	16	54	0.9063	145	15	6	64

Bold values are the highest values.

**Table 5:** Specificity, Gmean, F-score values for ELM, TLBO-ELM, Jaya-ELM and CJaya-ELM.

HN	ELM			TLBO-ELM			Jaya-ELM			CJaya-ELM		
	Spe	Gmean	F-score	Spe	Gmean	F-score	Spe	Gmean	F-score	Spe	Gmean	F-score
10	0.6923	0.7773	0.7721	0.7385	0.8462	0.8384	0.6462	0.7866	0.7716	0.6406	0.7833	0.7678
20	0.8141	0.8411	0.8406	**0.8571**	0.9112	0.9095	0.7714	**0.8589**	0.8540	0.7714	0.8589	0.8540
30	0.7632	0.8179	0.8160	0.8026	0.8871	0.8827	0.7237	0.8368	0.8280	0.7200	0.8347	0.8257
40	0.6974	0.7876	0.7818	0.7368	0.8556	0.8461	0.6579	0.8032	0.7874	0.6579	0.8032	0.7874
50	0.7879	0.8346	0.8332	0.8333	0.9045	0.9015	0.7424	0.8484	0.8409	0.7385	0.8462	0.8384
60	0.6765	0.7727	0.660	0.7206	0.8410	0.8310	0.6324	0.7828	0.7653	0.6324	0.7828	0.7653
70	0.7160	0.7934	0.7893	0.7531	0.8620	0.8542	0.6790	0.8129	0.7999	0.6750	0.8106	0.7972
80	0.6825	0.7645	0.7596	0.7302	0.8338	0.8265	0.6349	0.7726	0.7580	0.6349	0.7726	0.7580
90	0.7662	0.8131	0.8116	0.8052	0.8825	0.8788	0.7273	0.8331	0.8254	0.7237	0.8311	0.8232
100	0.7260	0.7813	0.7792	0.7671	0.8504	0.8459	0.6849	0.7981	0.7888	0.6849	0.7981	0.7888
110	0.6712	0.7819	0.7729	0.7123	0.8494	0.8364	0.6301	0.7938	0.7731	0.6250	0.7906	0.7692
120	0.7000	0.7937	0.7875	0.7375	0.8616	0.8513	**0.8625**	0.8112	0.7949	0.6625	0.8112	0.7949
130	0.7813	0.8261	0.8248	0.8281	0.8962	0.8934	0.7344	0.8387	0.8313	0.7302	0.8364	0.8287
140	0.6184	0.7141	0.7068	0.6579	0.7816	0.7701	0.5789	0.7281	0.7094	0.5789	0.7281	0.7094
150	0.7302	0.7962	0.7932	0.7778	0.8659	0.8610	0.6825	0.8061	**0.9151**	0.6774	0.8032	0.7917
160	0.8000	**0.8442**	**0.8430**	0.8462	**0.9143**	**0.9115**	0.7538	0.8577	0.8506	0.7538	0.8577	0.8506
170	0.7313	0.7869	0.7848	0.7761	0.8563	0.8522	0.6866	0.8001	0.7909	0.6818	0.7976	0.7878
180	0.6471	0.7150	0.7115	0.6912	0.7838	0.7777	0.6029	0.7270	0.7144	0.6029	0.7270	0.7144
190	0.7500	0.7906	0.7895	0.7941	0.8603	0.8576	0.7059	0.8058	0.7987	0.7015	0.8035	0.7961
200	0.6986	0.7808	0.7760	0.7397	0.8490	0.8410	0.6575	0.7952	0.7811	0.6575	0.7952	0.7811
210	0.6757	0.7475	0.7437	0.7162	0.8159	0.8090	0.6351	0.7630	0.7504	0.6301	0.7602	0.7470
220	0.8133	0.8195	0.8195	0.8533	0.8904	0.8896	0.7733	0.8417	0.8387	0.7733	0.8417	0.8387
230	0.7500	0.7982	0.7966	0.7969	0.8681	0.8650	0.7031	0.8103	0.8022	0.8413	0.8865	0.8853
240	0.6494	0.7399	0.7337	0.6883	0.8077	0.7974	0.6104	0.7553	0.7385	0.7403	0.8318	0.8262
250	0.8116	0.8001	0.8000	0.8551	0.8715	0.8713	0.7681	0.8202	0.8184	0.9063	0.8913	0.8911
260	0.7536	0.7949	0.7938	0.7971	0.8646	0.8618	0.7101	0.8107	0.8036	0.8551	0.8896	0.8889
270	0.7887	0.8214	0.8207	0.8310	0.8913	0.8891	0.7465	0.8392	0.8335	0.9143	0.9289	0.9288
280	0.7465	0.7782	0.7776	0.7887	0.8481	0.8459	0.7042	0.7958	0.7899	0.9437	0.9213	0.9210
290	0.6462	0.7271	0.7221	0.6923	0.7960	0.7883	0.6000	0.7361	0.7210	**0.9688**	**0.9356**	**0.9350**
300	**0.8143**	0.8165	0.8165	0.8571	0.8874	0.8869	0.7714	0.8361	0.8334	0.9143	0.9103	0.9103

Bold values are the highest values.

**Figure 6:**
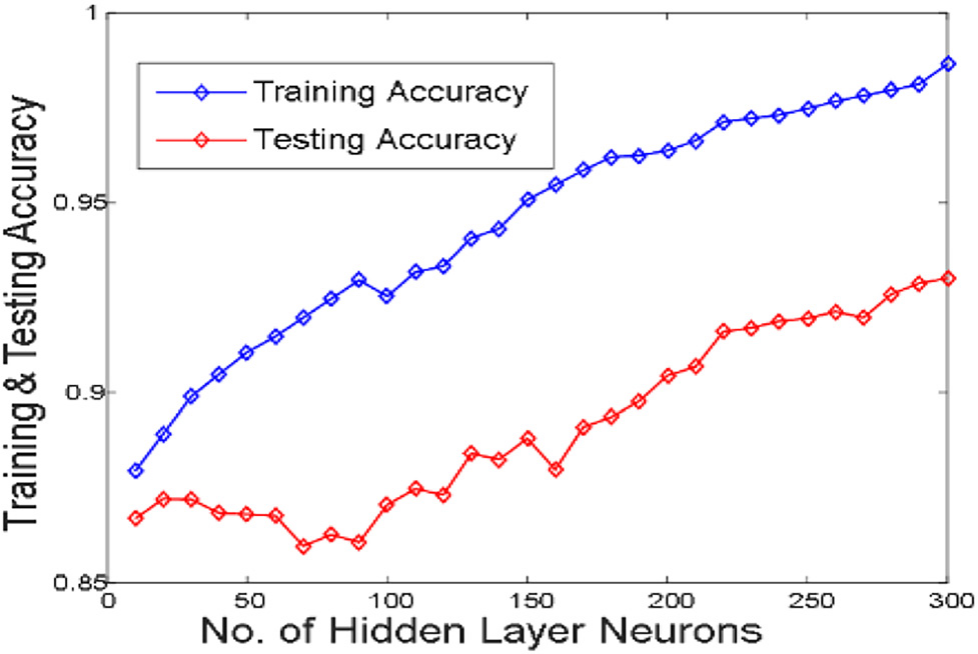
Hidden neurons versus training & testing accuracy (ELM).

**Figure 7:**
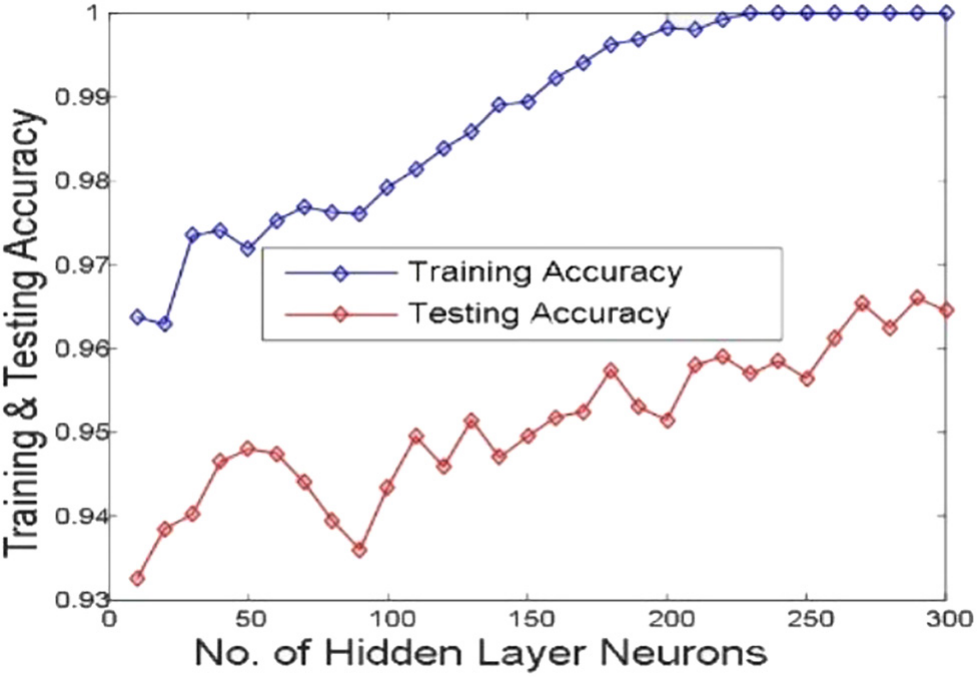
Hidden neurons versus training & testing accuracy (TLBO-ELM).

**Figure 8:**
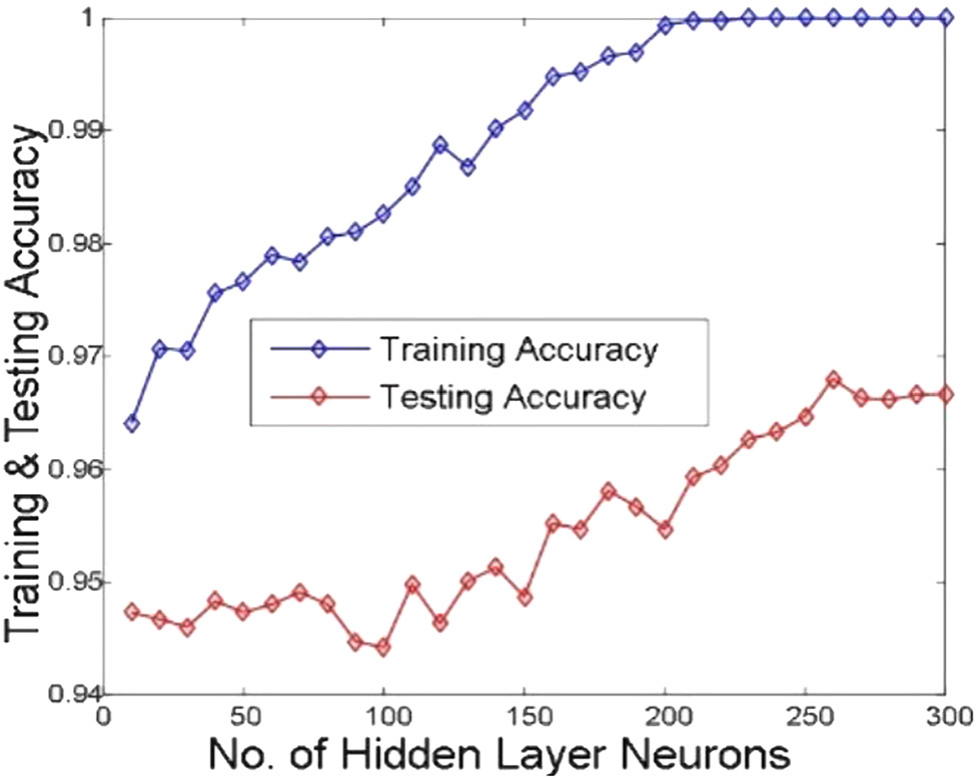
Hidden neurons versus training & testing accuracy (Jaya-ELM).

**Figure 9:**
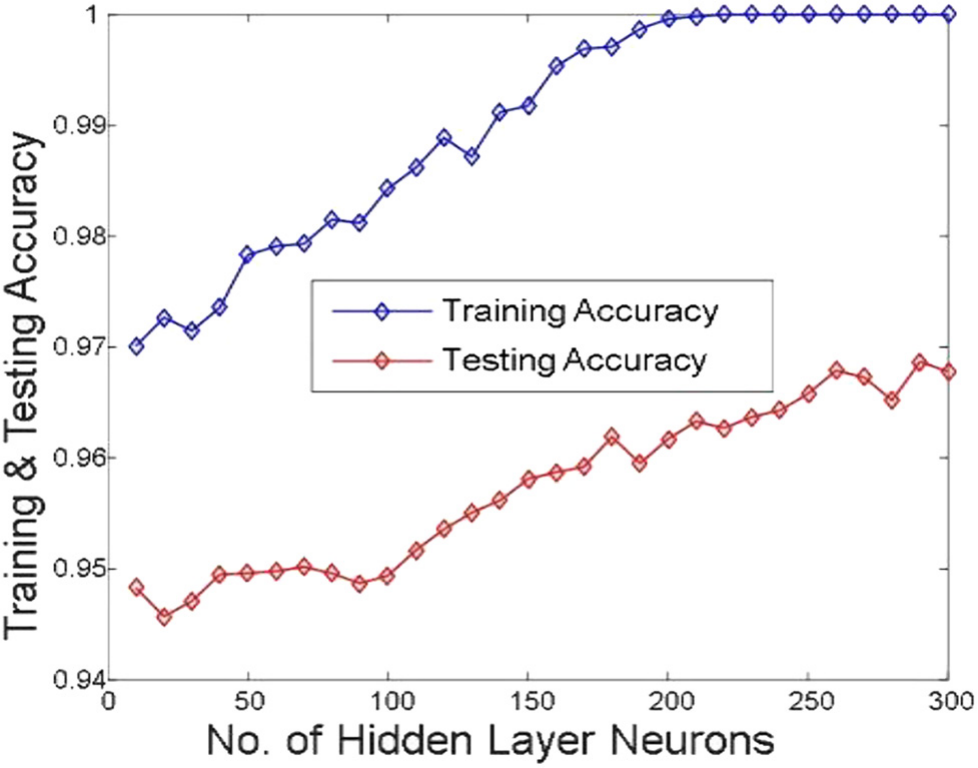
Hidden neurons versus training & testing accuracy (CJaya-ELM).

**Figure 10:**
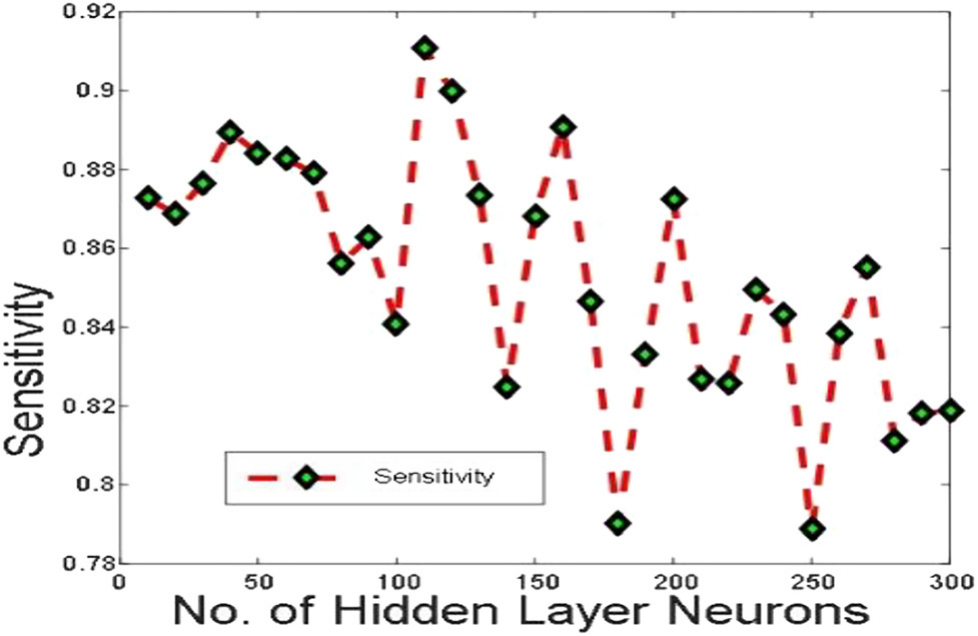
Sensitivity in ELM.

**Figure 11:**
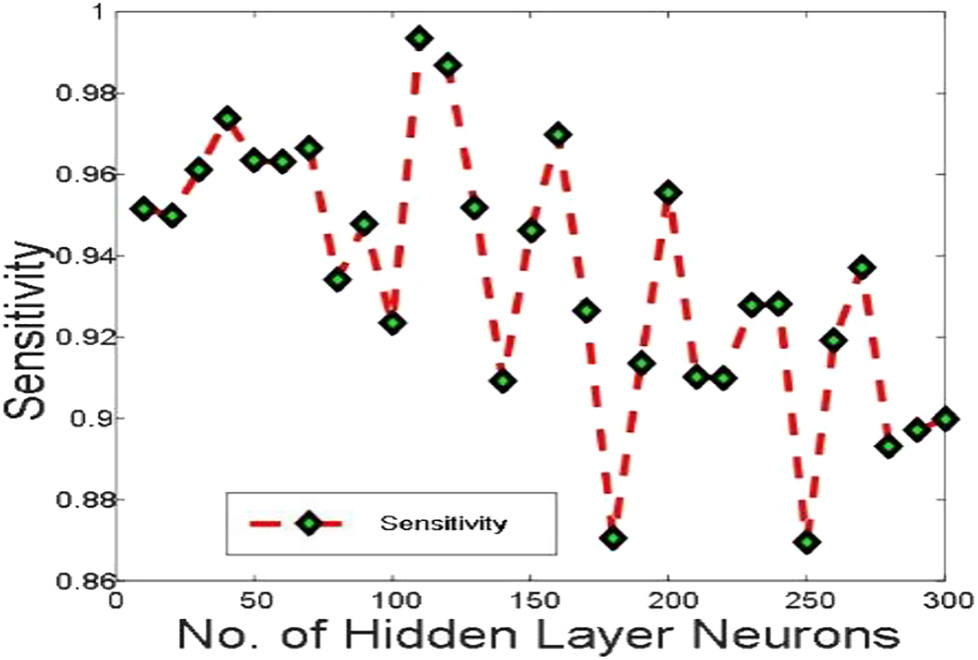
Sensitivity in TLBO-ELM.

**Figure 12:**
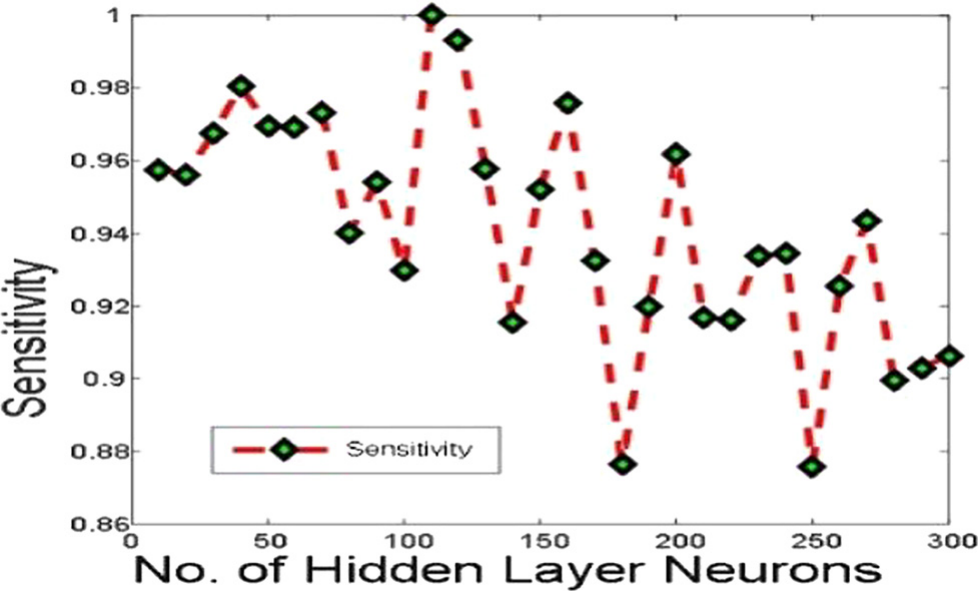
Sensitivity in Jaya-ELM.

**Figure 13:**
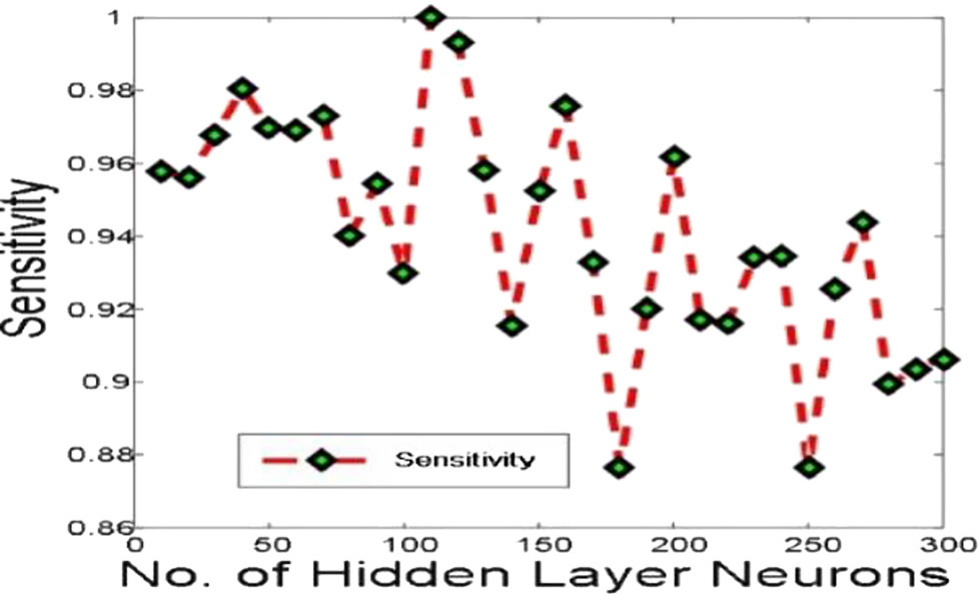
Sensitivity in CJaya-ELM.

**Figure 14:**
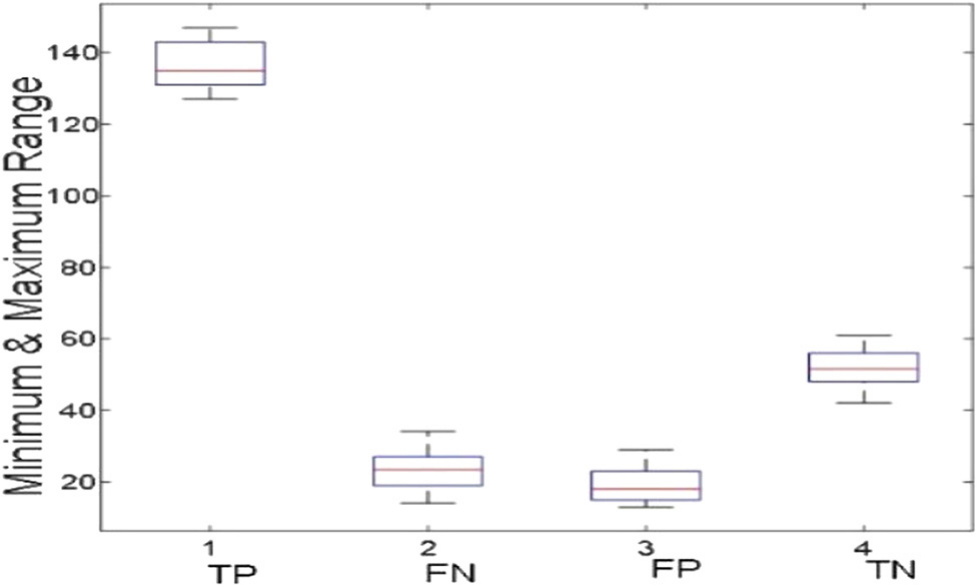
Boxplot (ELM).

**Figure 15:**
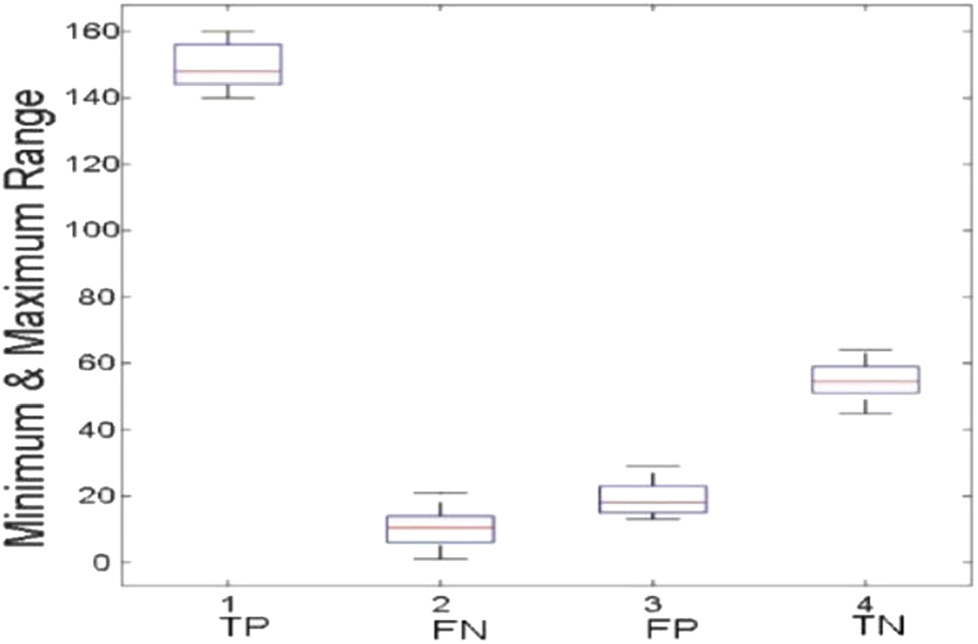
Boxplot (TLBO-ELM).

**Figure 16:**
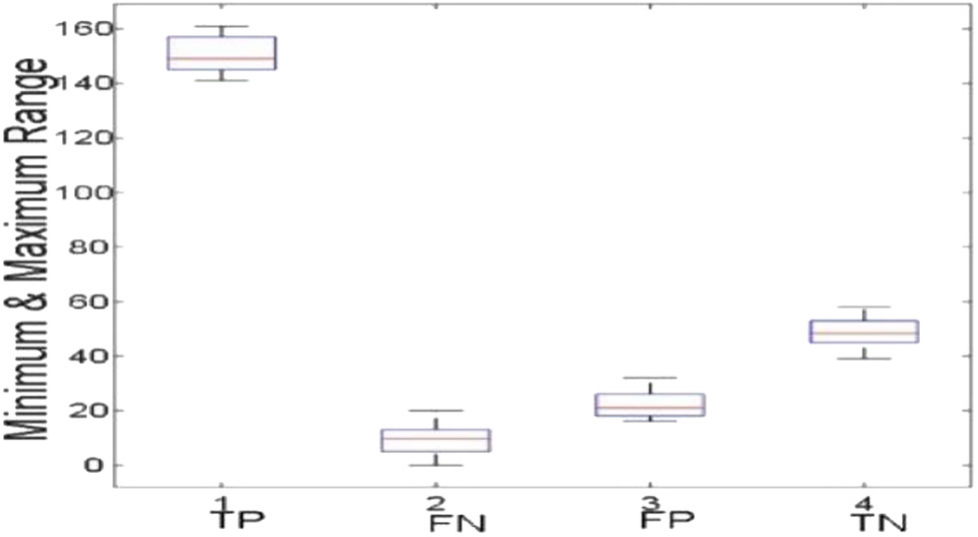
Boxplot (Jaya-ELM).

**Figure 17:**
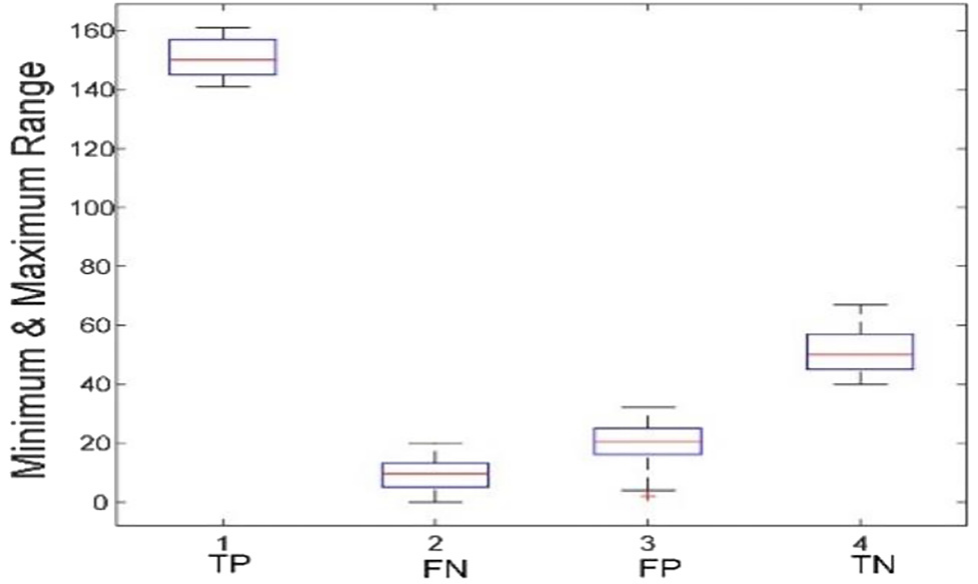
Boxplot (CJaya-ELM).

**Figure 18:**
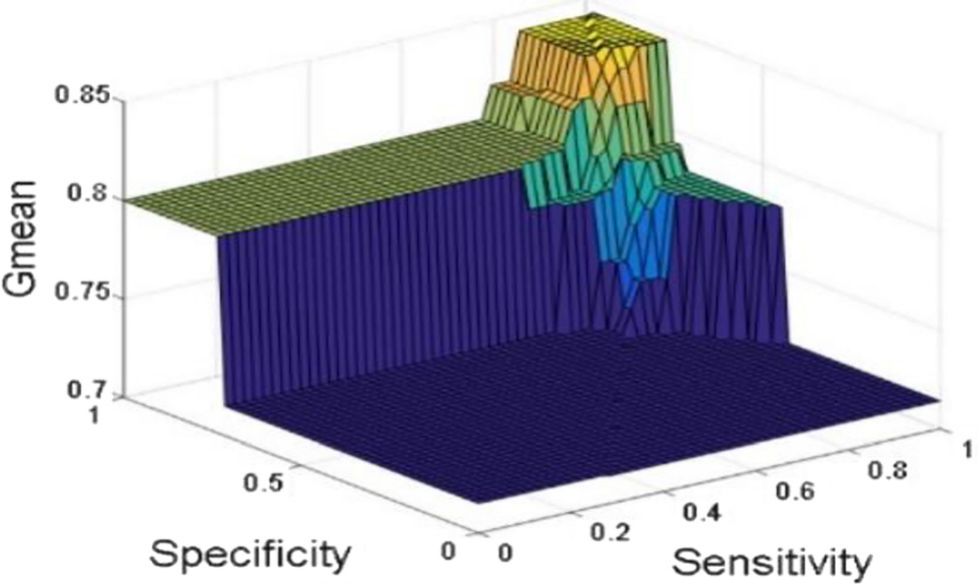
Sensitivity versus specificity versus Gmean (ELM).

**Figure 19:**
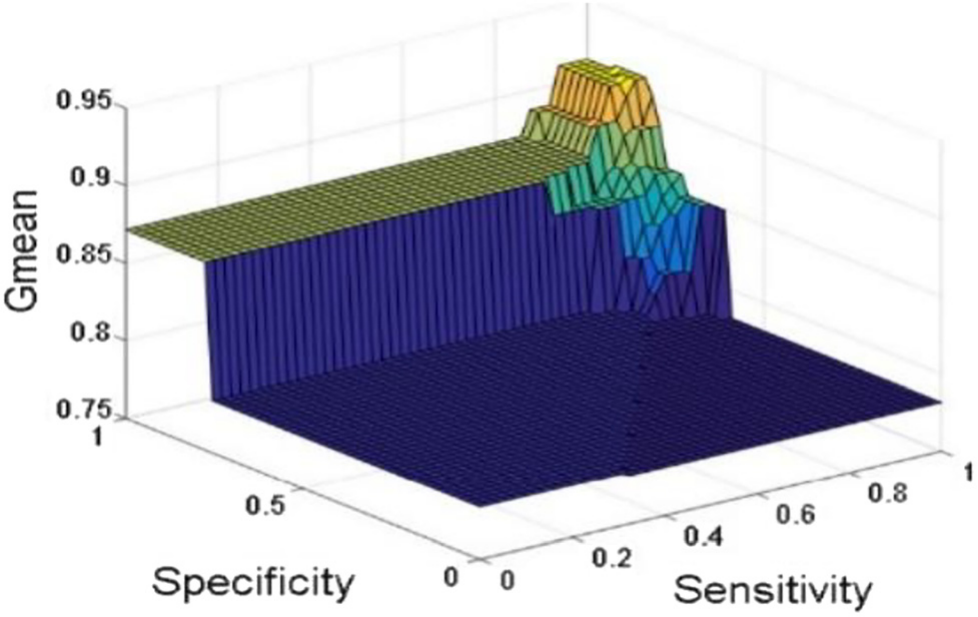
Sensitivity versus specificity versus Gmean (TLBO-ELM).

**Figure 20:**
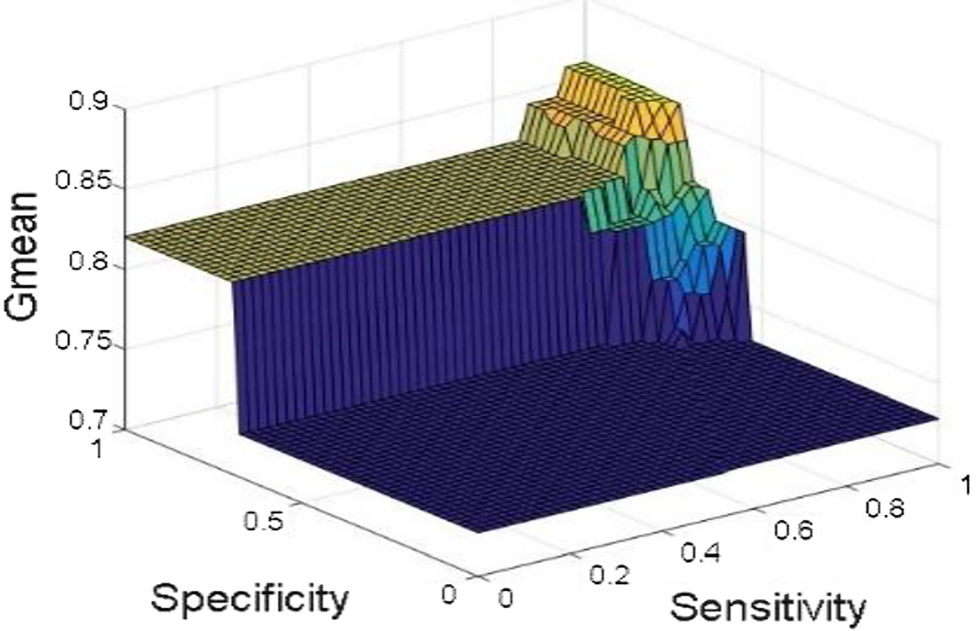
Sensitivity versus specificity versus Gmean (Jaya-ELM).

**Figure 21:**
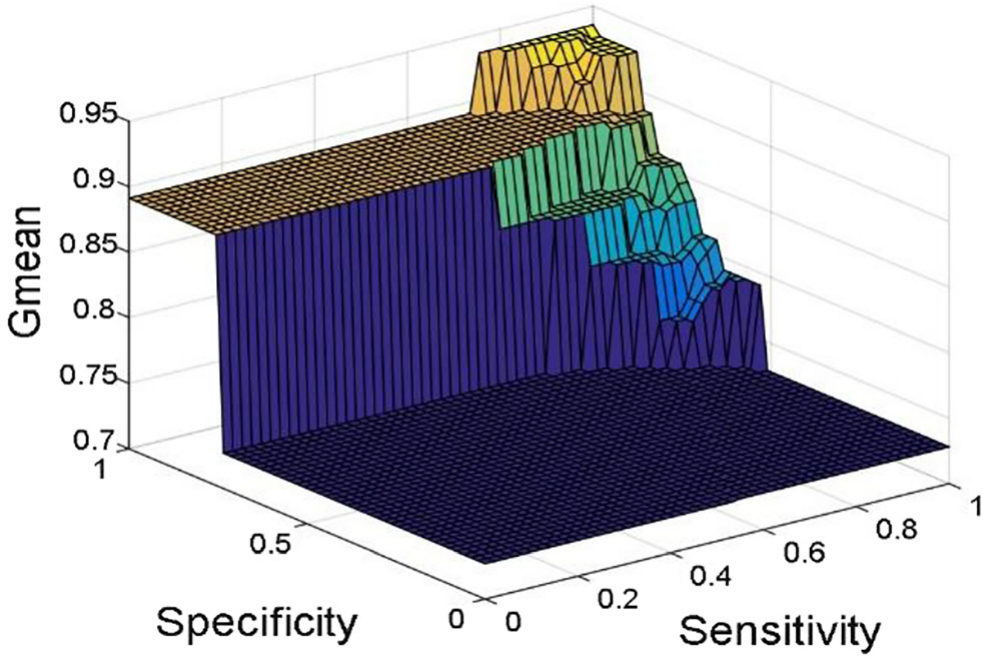
Sensitivity versus specificity versus Gmean (CJaya-ELM).

**Figure 22:**
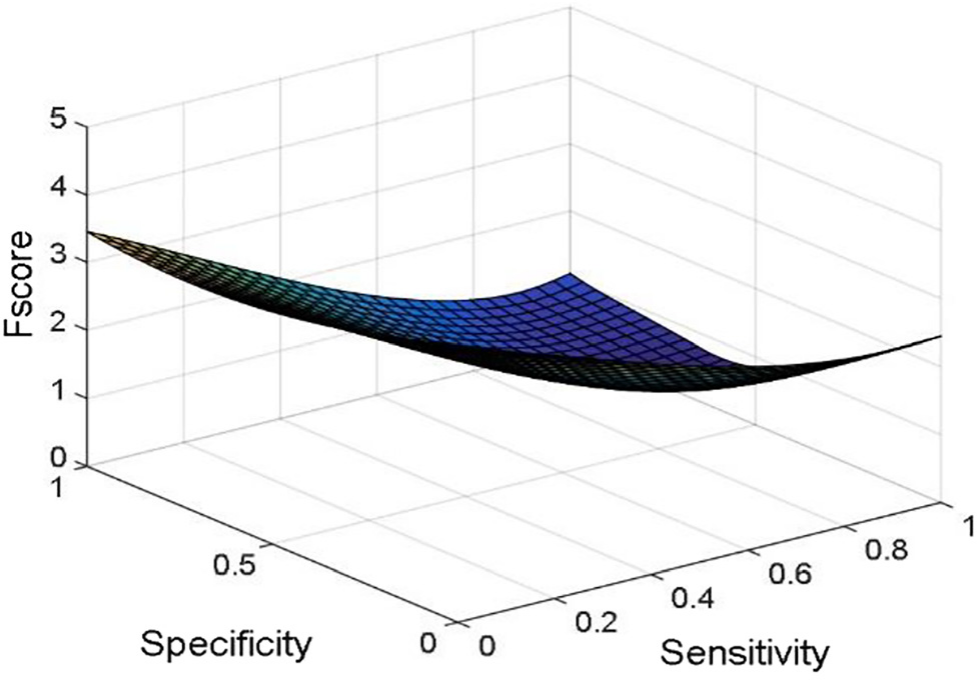
Sensitivity versus specificity versus F-score (CJaya-ELM).

**Figure 23:**
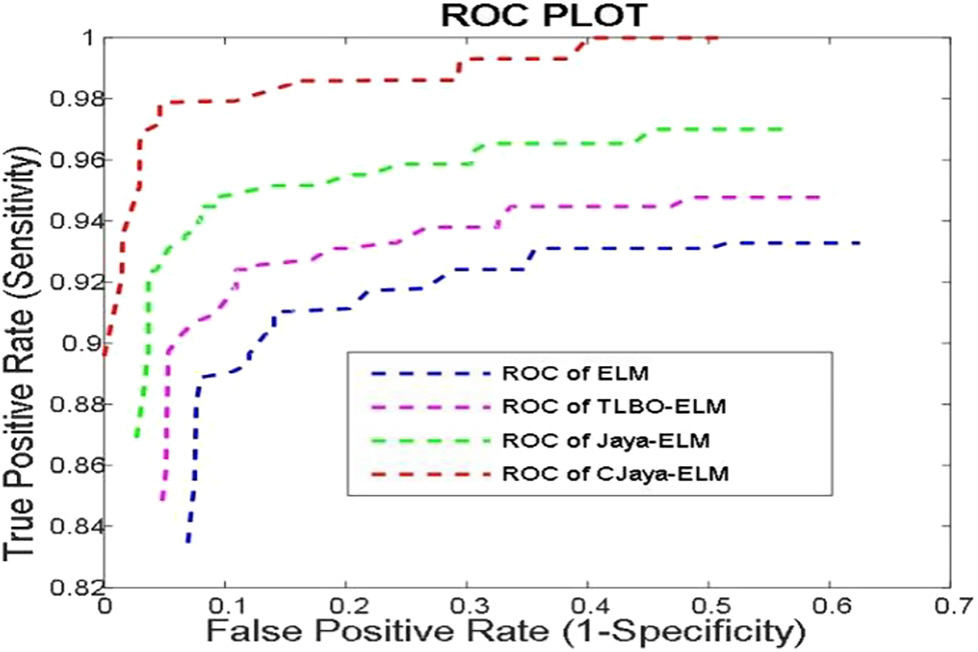
ROC of ELM, TLBO_ELM, Jaya_ELM and CJaya-ELM.

Figures 6–9 show training and testing accuracy of ELM, TLBO-ELM, Jaya-ELM and CJaya-ELM. Sensitivity w.r.t hidden neurons of all the ELM based models are shown in Figures 10–13 [Bibr j_jib-2019-0097_ref_011] [Bibr j_jib-2019-0097_ref_012]. Figures 14–17 [Bibr j_jib-2019-0097_ref_015] [Bibr j_jib-2019-0097_ref_016] give box plot for ELM, TLBO-ELM, Jaya-ELM and CJaya-ELM. Figures 18–21 [Bibr j_jib-2019-0097_ref_019] [Bibr j_jib-2019-0097_ref_020] present sensitivity versus specificity versus Gmean graphs for all the ELM, TLBO-ELM, Jaya-ELM and CJaya-ELM models whereas Figure 22 presents sensitivity versus specificity versus F-score graph of CJaya-ELM based model. Figure 23 displays the ROC curve of CJaya-ELM with AUC value.

Table 6 shows the Maximum training with testing accuracies of both MLP and ELM based models. Table 7 displays the AUC values of MLP, TLBO-MLP, Jaya-MLP, ELM, TLBO-ELM, Jaya-ELM and CJaya-ELM models.

**Table 6:** Maximum training (Tr_Acc) and testing (Ts_Acc) accuracies of MLP, TLBO-MLP, Jaya-MLP, ELM, TLBO-ELM, Jaya-ELM and CJaya-ELM.

Dataset	MLP		TLBO-MLP		Jaya-MLP		ELM		TLBO-ELM		Jaya-ELM		CJaya-ELM	
	Tr_Acc	Ts_Acc	Tr_Acc	Ts_Acc	Tr_Acc	Ts_Acc	Tr_Acc	Ts_Acc	Tr_Acc	Ts_Acc	Tr_Acc	Ts_Acc	Tr_Acc	Ts_Acc
Pima	0.8165	0.7278	0.8835	0.7600	0.917	0.7637	0.9865	0.8721	1	0.9675	1	0.968	1	0.9687

**Table 7:** AUC values of MLP, TLBO-MLP, Jaya-MLP, ELM, TLBO-ELM, Jaya-ELM and CJaya-ELM models.

Dataset	MLP	TLBO-MLP	Jaya-MLP	ELM	TLBO-ELM	Jaya-ELM	CJaya-ELM
Pima	0.6853	0.7053	0.8367	0.9302	0.9660	0.9680	0.9782

## 5 Result discussion

In this paper, Jaya optimization algorithm is used because it does not require any algorithm- specific parameter to be adjusted unlike PSO and DE optimization algorithms. Unlike TLBO, Jaya does not require two phases like teaching phase and learning phase. Still, Jaya algorithm is working with two random variables which may produce suboptimal result. These random numbers of Jaya algorithm are also generated by adopting a chaotic random number generator which not only produces optimal result but also improves the convergence speed and provides the better exploration of the search space without trapping in local optima. Here, mainly Chaos theory is integrated with Jaya algorithm to refine the quality of the best solution.

In this work, Jaya algorithm is modified by Chaos learning method which is termed as CJaya optimization algorithm. This CJaya algorithm optimizes the random parameters of ELM and called as CJaya-ELM model. Here, the CJaya-ELM model is proposed to classify the Pima Indian diabetes dataset. The obtained results are compared with MLP, TLBO-MLP, Jaya-MLP, CJaya-MLP, ELM, TLBO-ELM and Jaya-ELM. Here, the performance evaluating attributes viz. testing and training accuracy, confusion matrix, sensitivity, specificity, Gmean, F-score and ROC with AUC values are considered for evaluating the proposed model. The Table 3 gives a comparison of training and testing accuracy with respective hidden neurons of all the ELM-based models. From Table 3 and Figures 6–9 [Bibr j_jib-2019-0097_ref_007] [Bibr j_jib-2019-0097_ref_008], it is clearly visualized that the proposed CJaya-ELM model acquires more testing accuracy (0.9783) with a smaller number of hidden neurons (230) in comparison to other ELM-based models. Table 6 displays the comparison between the maximum training and testing accuracy of all the ELM based models and MLP based models. This comparison reveals the superiority of CJaya-ELM model over other models. The AUC values of all the models are compared in Table 7.

Tables 4, 5 and Figures 10–13 [Bibr j_jib-2019-0097_ref_011] [Bibr j_jib-2019-0097_ref_012] clearly show that CJaya-ELM model has more sensitivity (1) and specificity (0.9688) with respect to other ELM-based models. If sensitivity of the model will be more then positive samples are well classified and if specificity will be more then negative samples are well classified. This indicates that CJaya-ELM classifier classifies both the positive and negative samples more accurately than other models. Moreover, the superiority of CJaya-ELM model can be seen from the 3-D graphs of Sensitivity versus Specificity versus Gmean through Figures 18–21 [Bibr j_jib-2019-0097_ref_019] [Bibr j_jib-2019-0097_ref_020] as its sensitivity and specificity are higher than other models.

As ROC is a graphical representation between sensitivity and specificity. So, the Area under ROC curve i.e. AUC value determines an aggregate evaluation of performance across all possible classification thresholds. The ROC graph of Figure 23 and Table 7 show the AUC values of the different models. The highest AUC value (0.9782) shows the superiority of CJaya-ELM model over other models.

The box plots (Figures 14–17 [Bibr j_jib-2019-0097_ref_015] [Bibr j_jib-2019-0097_ref_016]) show the TP, TN value exceed FP and FN value in CJaya-ELM model than other ELM-based models which indicates that positive samples and negative samples are correctly classified.

The above observations reveal that basic ELM based models like ELM, TLBO-ELM, Jaya-ELM and CJaya-ELM are superior classifiers in comparison to MLP based models such as Jaya-MLP, TLBO-MLP and CJaya-MLP. Apart from that, when basic ELM based models are compared with the presented CJaya-ELM model, it is proved that CJaya-ELM is significantly better.

## 6 Conclusions

In this work, CJaya-ELM is presented for classification of Pima diabetes dataset. Other two ELM based models such as basic ELM, TLBO-ELM and three MLP based models like MLP, Jaya-MLP and TLBO-MLP are also discussed and compared. The proposed model is evaluated through a series of empirical studies. In order to perform an unbiased comparison among all the models, many performance measuring attributes like testing accuracy, training accuracy, confusion matrix, sensitivity, specificity, Gmean, F-score and ROC with AUC values are considered. Here, ELM is integrated with CJaya algorithm to make the classifier more robust. The outcomes prove that CJaya-ELM can successfully handle the ill-condition problem and gives better performance in comparison with ELM, TLBO-ELM, MLP, Jaya-MLP and TLBO-MLP. This study concludes that the proposed CJaya-ELM model efficiently classifies the diabetic data and helps in identifying the diabetes in pregnant women.
